# Making the most of your pollinators: An epiphytic fig tree encourages its pollinators to roam between figs

**DOI:** 10.1002/ece3.7488

**Published:** 2021-03-31

**Authors:** Siti Khairiyah Mohd Hatta, Rupert J. Quinnell, Abd Ghani Idris, Stephen G. Compton

**Affiliations:** ^1^ Faculty of Biological Sciences University of Leeds Leeds UK; ^2^ Faculty of Applied Sciences Universiti Teknologi MARA Shah Alam Malaysia; ^3^ Faculty of Science and Technology Universiti Kebangsaan Malaysia Bangi Malaysia

**Keywords:** dioecy, evolutionary constraints, *Ficus*, inflorescence design, pollination, vicarious selection

## Abstract

*Ficus* species are characterized by their unusual enclosed inflorescences (figs) and their relationship with obligate pollinator fig wasps (Agaonidae). Fig trees have a variety of growth forms, but true epiphytes are rare, and one example is *Ficus deltoidea* of Southeast Asia. Presumably as an adaptation to epiphytism, inflorescence design in this species is exceptional, with very few flowers in female (seed‐producing) figs and unusually large seeds. Figs on male (pollinator offspring‐generating) trees have many more flowers. Many fig wasps pollinate one fig each, but because of the low number of flowers per fig, efficient utilization by *F. deltoidea*'s pollinators depends on pollinators entering several female figs. We hypothesized that it is in the interest of the plants to allow pollinators to re‐emerge from figs on both male and female trees and that selection favors pollinator roaming because it increases their own reproductive success. Our manipulations of *Blastophaga* sp. pollinators in a Malaysian oil palm plantation confirmed that individual pollinators do routinely enter several figs of both sexes. Entering additional figs generated more seeds per pollinator on female trees and more pollinator offspring on male trees. Offspring sex ratios in subsequently entered figs were often less female‐biased than in the first figs they entered, which reduced their immediate value to male trees because only female offspring carry their pollen. Small numbers of large seeds in female figs of epiphytic *F. deltoidea* may reflect constraints on overall female fig size, because pollinator exploitation depends on mutual mimicry between male and female figs.

## Graphical abstract

*Blastophaga* sp. pollinators routinely enter several figs of both sexes and generated more seeds per pollinator on female trees and more offspring on male trees. Pollinator offspring sex ratios in subsequently entered figs (circles) were often less female‐biased than in the first figs they entered (squares).

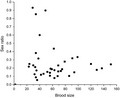

## 1 INTRODUCTION

Many plant families are characterized by the way their flowers are grouped together into inflorescences. Design features of inflorescences, including the size and number of flowers they contain and how they are arranged, can influence pollination rates, levels of seed production, and the numbers of seeds that are subsequently dispersed away from parent plants (Harder & Prusinkiewicz, 2013; Ishii et al., 2008; Wyatt, 1982). Among the likely trade‐offs in inflorescence design is the relationship between the numbers of flowers in an inflorescence and the size of the flowers (Burd, 2008; Worley & Barrett, 2001). As functional units central to the sexual reproductive success of a plant, external selection pressures influencing inflorescence design vary with the specific behavior of pollinators (Endress, 2010). These selection pressures may also act differently on male and female reproductive functions, driving differences between male, female, and bisexual inflorescences (Emms et al., 1997; Fishbein & Venable, 1996; Lau et al., 2008). After pollination, the behavior of any animals responsible for the dispersal of the seeds may also have a significant influence on inflorescence design, because the size and location of inflorescences largely determine infructescence characteristics and which animals are attracted to them (Fleming & Kress, 2011).

The genus *Ficus* (Moraceae) is characterized by its unusual enclosed protogynous inflorescences (figs, also called syconia). All *Ficus* species are pollinated by one or several congeneric species of fig wasps (Agaonidae, Hymenoptera), whose behavior and anatomy are strongly influenced by the structure of their host figs. The insides of figs are lined with tiny flowers, and entry of pollen is only possible via a narrow slit called the ostiole (Thorogood et al., 2018). Although the basic structure of figs is conserved throughout the genus, within the over 800 described species there is considerable variation in where the inflorescences are produced (underground, from the trunk, or among the leaves), whether both male and female flowers are present, whether male flowers are scattered or aggregated, how many flowers are present, the external diameter of the figs, and whether they ripen to offer a reward to frugivores (Berg & Corner, 2005). The overall structure of figs, and the co‐adaptive features they share with their pollinators, has apparently remained essentially unchanged for tens of millions of years, and much of the current variation in the size and location of figs may reflect adaptations to facilitate the attraction of different groups of vertebrate frugivores (Compton et al., 2010).

Foundress female fig wasps enter the figs in order to lay their eggs inside the uniovulate female flowers located on their inner surface. Each species of fig wasp is typically associated with a single species of fig tree, although isolated examples of one fig wasp pollinating more than one species on a regular basis have been recorded (Cornille et al., 2012). The specificity of the association is achieved mainly through the release of species‐ and developmental stage‐specific attractant volatiles at the time when the female flowers are ready to be pollinated (van Noort et al., 1989; Wang et al., 2013). When passing through the ostiole, pollinators lose their wings and parts of their antennae (Kjellberg et al., 2005) and ostiole shape and size are reflected in the head shapes of pollinators (van Noort & Compton, 1996). Once inside, foundresses attempt to oviposit into the ovules of the fig flowers by inserting their ovipositors through the stigma and then along the length of the styles. For their larvae to develop successfully, the foundresses must be capable if initiating gall development in the ovules where their eggs are laid. Initial gall development is stimulated by their mothers, with galled ovules expanding rapidly to occupy the limited space available within each fig (Ghana et al., 2015). Pollinator larvae develop singly inside the galled ovules.

Although the relationship between fig trees and fig wasps is characterized as an obligate mutualism, about half of all fig tree species are functionally dioecious, with only one sex supporting the development of pollinator offspring. In these species, the relationship between pollinators and male trees is directly mutualistic (with fig wasps developing in figs and then transporting pollen from their hosts), but if a fig wasp enters a fig on a female tree she generates seeds, but cannot herself reproduce. This is a consequence of the long styles of flowers in the female figs and the structure of their stigmas, which together prevent the wasps from galling the ovules and laying their eggs (Kjellberg et al., 2014). Fig wasps continue to enter female figs because male and female figs at the developmental stage when they require pollination present similar cues to the pollinators as a result of vicariant selection (Grafen & Godfray, 1991). In particular, conspecific male and female receptive figs release broadly similar blends of attractant volatiles (Proffit et al., 2020). After entry into female figs, foundresses of actively pollinating species (Kjellberg et al., 2001) remove pollen from their pollen baskets and deposit it on the stigmas, despite being unable to oviposit. This reflects another example of vicariant selection acting on those foundresses that entered male figs and had offspring, because all foundresses that enter female figs fail to reproduce (Raja et al., 2008a). Pollinators that transport pollen passively lack direct control of pollen deposition, but are likely to shed more pollen the longer they remain active inside a fig, searching in vain for suitable oviposition sites.

Many foundresses never re‐emerge from the first receptive figs they enter, but the frequency of pollinator foundress re‐emergence after entering a fig varies between species (Gibernau et al., 1996; Suleman et al., 2013) and may be more common among pollinators of dioecious than monoecious fig trees (Moore et al., 2003). Re‐emergence depends on the figs having their ostioles open for an extended period and for the pollinator foundresses to exhibit appropriate behavior. Natural selection will favor a willingness to re‐emerge if the foundresses that do so have more offspring than those that choose to remain in the first fig they enter and lay all their eggs there (Suleman et al., 2013). Only one offspring can develop in a single flower (Jousselin et al., 2002) and if the combined eggs loads of the foundresses that enter a fig are more than the total number of flowers available, then oviposition site limitation is inevitable. This means that if the egg load of a single female pollinator is larger than the number of female flowers present in a single fig, then re‐emergence behavior would always be favored, because even if only a small proportion of re‐emerged females successfully enter more than one fig they can still produce more offspring than if they stayed in the first fig (Gibernau et al., 1996). The benefits of re‐emergence will also vary according to local environmental factors (Gibernau et al., 1996), including the likelihood of predation by ants (Bain et al., 2014). Re‐emergence may also be flexible and responsive to aggression between ovipositing foundresses (Moore & Greeff, 2003) and the extent of competition for the limited number of oviposition sites available in any one fig (Moore et al., 2003).

Fig wasp offspring sex ratios are female‐biased, but as the number of foundresses sharing a fig increases, the bias toward females often declines (Peng et al., 2014). This effect is generated largely by females laying more male eggs at the start of an oviposition sequence in combination with increasing competition for oviposition sites resulting in fewer offspring per foundress in figs shared with others (Greeff & Newman, 2011; Raja et al., 2008a). In a *Ficus* species where foundresses can pollinate and lay eggs in several figs, it was found that under glasshouse conditions brood sizes were larger in the first figs they entered and that later broods contained a higher proportion of male offspring (Greeff et al., 2020; Moore et al., 2003; Raja et al., 2008a). Only female fig wasps carry pollen, so less strongly female‐biased sex ratios are less productive for male plants, but this may be partially compensated for by more pollen per fig wasp being carried by the smaller number of female offspring sharing a fig (Kjellberg et al., 2014).

The dioecious *F. deltoidea* is highly unusual among *Ficus* species in that the figs produced by female plants contain only small numbers of flowers, and in some varieties just a single flower (Corner, 1969). Its seeds are also unusually large, a feature that may be linked to the ability of some varieties to grow as true epiphytes (Corner, 1969). This growth form is rare among fig trees (in contrast to the many hemi‐epiphytic stranglers that start as epiphytes, but eventually send roots to the ground).

One variety of *F. deltoidea (var. angustifolia)* occurs regularly as an epiphyte in oil palm plantations in peninsular West Malaysia (Mohd Hatta, 2019). Seed production in *Ficus* species is often pollinator‐limited (Bronstein, 1988; Compton et al., 1994), and a large majority of figs of *F. deltoidea var. angustifolia* in Malaysian oil palm plantations abort after failing to be pollinated (Mohd Hatta, 2019). The unusually small number of flowers in each female fig of *F. deltoidea* means that each foundress that enters a fig carries more pollen that is required to pollinate all of its flowers, and observations of one variety of *F. deltoidea* with few flowers in its female figs found that the stigmas of each flower had received multiple pollen grains (Jousselin & Kjellberg, 2001). The plant would therefore be making inefficient use of its pollinators if each foundress entered just one fig because it had been trapped inside. Having female figs that allow foundresses to re‐emerge from receptive figs and go on to pollinate further figs would clearly be advantageous for female plants. However, to modify pollinator behavior, male plants must also allow foundress females to re‐emerge from their figs, and those females must achieve greater reproductive success than females that lay their entire egg load in a single fig, despite the risks associated with needing to walk between figs. Only then will natural selection favor pollinator re‐emergence. It can only take place on male trees because that is where the fig wasps can successfully reproduce, but its effects can provide vicariant benefits for the female trees, as is the case with the apparent mutual mimicry in attractant volatile production by male and female figs (Grafen & Godfray, 1991). This is analogous to the selection for active pollen dispersal in female figs of other *Ficus* species, which occurs despite the behavior being of no benefit to the individual foundresses (Raja et al., 2008a).

We sought to understand the pollination biology of one of the varieties of *F. deltoidea* that has few flowers in its female figs. The specific questions we asked were (1) Do foundresses re‐emerge from figs and do rates of emergence differ between male and female figs? (2) How many figs can a single foundress enter? (3) How many seeds are generated in female figs and do pollination rates per fig decline when a foundress enters additional figs? And (4) How many offspring do foundresses generate in their first and subsequent male figs, do females that re‐emerge produce more offspring, and do their offspring sex ratios change?

## 2 MATERIALS AND METHODS

### 2.1 Study site and study species

The study was carried out in Banting district, Selangor, West Malaysia, between April and July 2017. Banting has a tropical climate with average daily minimum temperatures ranging from 24.0 to 25.2°C, and maximum temperatures ranging between 30.9 and 32.5°C, with little seasonal variation. The average monthly precipitation is 144.3 mm. Our 2.1 ha oil palm plantation study site held about 285 oil palms that had been planted in 2001 on a peat soil. The oil palms at Banting supported 113 epiphytic individuals of *F. deltoidea var. angustifolia,* with usually a single plant per trunk. Leaf and fig production was asynchronous both within plants and across the population as a whole, and figs were present on the plants more or less continuously throughout the year (Mohd Hatta, 2019). The figs on female trees contained only 3–6 female flowers. Although the male and female figs had a similar small size at the developmental stage when they were attractive to pollinators (around 4 mm in diameter), the male figs contained an average of 143 female flowers. In addition, the male figs also contained up to 20 or more bistaminate male flowers (Mohd Hatta, 2019).

The specificity of the pollinators that service different varieties of *F. deltoidea* has not been clearly established. The only formally recognized pollinator is *Blastophaga quadrupes* Mayr, which was recorded from an unconfirmed variety of *F. deltoidea* in Java. In Peninsular Malaysia, the three varieties commonly present as epiphytes in oil palm plantations each have morphologically distinct *Blastophaga* spp. pollinators (Mohd Hatta, 2019). Here, we refer to the pollinator of *F. deltoidea var. angustifolia* as *Blastophaga* sp. It is a passive pollinator that transports pollen on its general body surface. Unusually for a *Ficus* species, no nonpollinating fig wasps were found in the figs of *F. deltoidea* at our study site and elsewhere in Malaysia (Mohd Hatta, 2019).

### 2.2 Natural pollination rates

The behavior of *Blastophaga* sp. in the plantation was recorded using 200 haphazardly collected figs from 21 male and 10 female trees, each of which was located on a different oil palm trunk. The figs were at early C phase (sensu Galil & Eisikowitch, 1968), the stage of development when the figs contain galled ovules (male trees) or developing seeds (female trees) and the bodies and wings of foundress females that died in the figs were also present. Seed or gall development confirmed that a foundress had entered the figs, and the presence of wings indicated that the fig had been the first one to be entered by at least one of the foundresses. The development of figs where no foundress was present indicated that one or more foundresses had entered, but had then re‐emerged. When the body of a foundress was present, the position and orientation of its head were recorded. If the body was in the central cavity of the fig cavity, they had potentially laid eggs or pollinated the ovules. Foundresses in the ostioles with their heads facing toward the center of the figs had died during their attempt to enter the fig.

### 2.3 Experimental manipulations

The behavior of individual foundresses on male and female figs was assessed by introducing individual *Blastophaga* sp. females into netting bags surrounding groups of 4–15 adjacent receptive figs. The figs had been bagged earlier (at A phase sensu Galil & Eisikowitch, 1968) to prevent prior entry by pollinators. One to two weeks later, mature male figs (at D phase sensu Galil & Eisikowitch, 1968) were collected locally just before the fig wasps were expected to emerge and placed in mesh‐covered containers to let the female fig wasps emerge naturally. Shortly after they emerged, a single foundress was placed in each bag using a fine brush. The bag was then closed again to prevent entry by other pollinators. In total, 63 bags were placed in 11 female trees and 63 bags in 10 male trees.

Six weeks later, the bags were opened to record how many developing figs were present, and we recorded the numbers of fig wasp offspring and seeds they contained. Pollinators had entered at least one fig in 30 of the bags on 6 male trees and 38 of the bags on 8 female trees. The first fig entered by each foundress was identified by the presence of the wings in the ostiole, but the precise sequence of entry by each wingless foundress into the subsequent figs could not be determined. The counts nonetheless allowed comparisons of the numbers of seeds generated by a single foundress in their first and subsequent figs and also the size of the clutches present. Total offspring sex ratios for a foundress could not be calculated if any offspring had emerged from one or more of the figs where they had oviposited, reducing our sample sizes accordingly.

### 2.4 Data analysis

Statistics were performed in R (1.0.153) and SPSS Statistics 20. The frequency of figs entered by a single foundress was analyzed using a chi‐square test. The total brood size for foundresses that entered different numbers of figs, total brood size of first entered and subsequent male figs, and total seed production in the first and subsequent female figs entered were analyzed using generalized linear models (GLMs) with the Poisson error distributions. If overdispersion occurred, quasi‐Poisson errors were used for counts of seeds and numbers of offspring. Offspring sex ratios of emerged and nonemerged foundresses were analyzed using generalized linear models (GLMs) with a Gaussian error and offspring sex ratios in the first and subsequently entered figs were compared using generalized linear models (GLMs) with a quasi‐binomial error distribution and logit link. Spearman's rank correlations were used when examining the relationship between brood size and sex ratio in the experimental figs.

## 3 RESULTS

### 3.1 Foundress numbers in a natural population

Almost half of the figs in Banting plantation that had galls or seeds present had no remains of foundresses inside. This showed that pollinator foundresses routinely re‐emerge from both male and female figs and that counts of their bodies in the figs indicate only the minimum number of foundresses that had entered the figs. Maxima of six and four foundresses remained in male figs and female figs, respectively. There was minor variation between trees in the proportion of figs where all the foundresses had re‐emerged, but all had some figs where no foundresses remained (in male figs, proportion with no foundresses remaining = 0.38, range = 0.43–0.80, *n* = 200 figs from 21 trees; in female figs, proportion with no remaining foundresses = 0.50, range = 0.25 –1.00, *n* = 200 figs from 10 trees). These values do not include pollinators that had apparently become trapped in the ostiole while attempting to enter the figs.

### 3.2 Entry and emergence after experimental foundress introductions

Evidence of the entry of the lone foundresses that had been introduced into the fine nylon mesh bags was provided by the development of galls in male figs and seeds in female figs. The figs of both sexes entered by wingless foundresses were usually located less than 6 cm from a fig where wings were present in its ostiole, showing that the foundresses had not walked far from the first figs they had entered. Wings were only detected in the ostioles of a single fig, indicating that they were consistently lost in the first figs entered.

**FIGURE 1:**
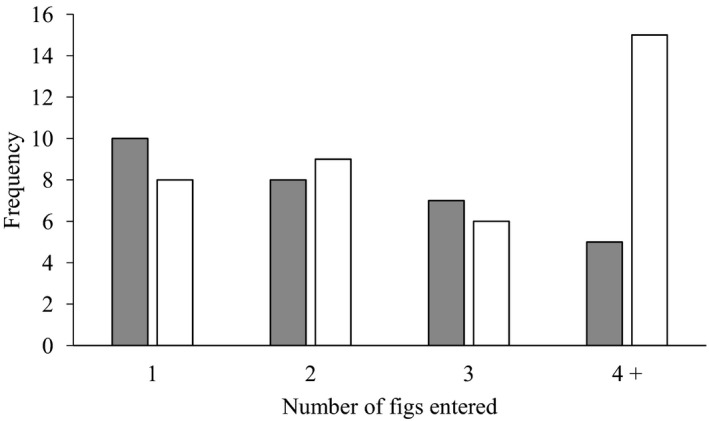
The number of times that single *Blastophaga* sp. foundresses entered one, two, three, or more figs. Solid bars = figs on male trees; open bars = figs on female trees

Among the 68 introduced foundresses that entered at least one fig (30 foundresses on male trees and 38 on female trees), 50 (73.53%) entered more than one fig with a maximum of four male figs and six female figs entered by the single foundresses (Figure 1). The likelihood of re‐emergence from the first fig was not significantly higher in female figs (79.0%) than male trees (66.7%) (chi‐square, *χ* ^2^ = 1.30, *df* = 1, *p* > .05). The total numbers of male or female figs entered by the introduced females also did not differ significantly (chi‐square, *χ* ^2^ = 4.48, *df* = 3, *p* > .05), but over twice as many foundresses entered four or more figs if the figs were female (Figure 1).

### 3.3 Pollination of female figs

Foundresses that were entering their first figs (as shown by leaving their wings in the ostiole) successfully pollinated all the flowers that were present (Mean ± *SE* seeds = 4.40 ± 0.10, *n* = 25 figs). Figs that they entered subsequently did not always have all their flowers pollinated (mean ± *SE* seeds = 3.87 ± 0.16, *n* = 47), but variation in the numbers of flowers present meant there was no significant difference in the numbers of flowers pollinated by the foundresses in their first and subsequent figs (GLM with the Poisson error, *χ* ^2^ = 0.38, *df* = 1, *p* > .05).

### 3.4 Pollinator offspring in male figs

**FIGURE 2:**
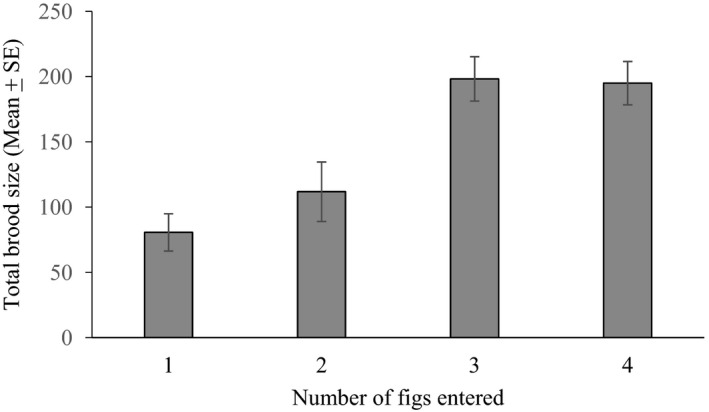
Total brood sizes of *Blastophaga* sp. foundresses that had entered different numbers of figs on male trees

There was a large difference in the total brood sizes achieved by *Blastophaga* sp. foundresses that re‐emerged and produced offspring in two or more figs and those that only produced offspring in the first figs they entered, with the former generating roughly twice the number of offspring (Figure 2, Table 1). Total brood sizes of females that laid all their eggs in a single fig ranged from 42 to 137 (mean ± *SE* = 80.70 ± 14.24, *n* = 6), whereas re‐emerging females had total brood sizes that ranged from 26 to 236 (mean ± *SE* = 161.42 ± 16.65, *n* = 12) (GLM, *χ* ^2^ = 100.15, *df* = 1, *p* < .05). There was also a significant difference in their combined brood sizes depending on the total number of figs they entered (GLM, *χ* ^2^ = 348.35, *df* = 3, *p* < .001) with the maximum number of offspring generated among foundresses that had entered three or four figs (Figure 2).

**TABLE 1:** Individual variation in the extent of *Blastophaga* sp. re‐emergence and fecundity from figs of *Ficus deltoidea* var. *angustifolia*

Foundress	No. of figs entered	Re‐emerged?	Female offspring	Male offspring	Offspring sex ratio	Total brood size
1	1	No	77	9	0.10	86
2	1	No	80	14	0.15	94
3	1	No	110	27	0.20	137
4	1	No	40	6	0.13	46
5	1	No	57	22	0.28	79
6	1	No	37	5	0.12	42
7	2	Yes	101	60	0.37	161
8	2	Yes	88	41	0.32	129
9	2	Yes	106	22	0.17	128
10	2	Yes	77	38	0.33	115
11	2	Yes	20	6	0.23	26
12	3	Yes	184	33	0.15	217
13	3	Yes	113	64	0.36	177
14	3	Yes	184	52	0.22	236
15	3	Yes	140	23	0.14	163
16	4	Yes	182	26	0.13	208
17	4	Yes	99	63	0.39	162
18	4	Yes	147	68	0.32	215
Total	1–4		1,842	579	0.24	2,421

Note

Only foundresses where all the offspring could be sexed are included. Offspring sex ratios are given as the proportion of males.

**FIGURE 3:**
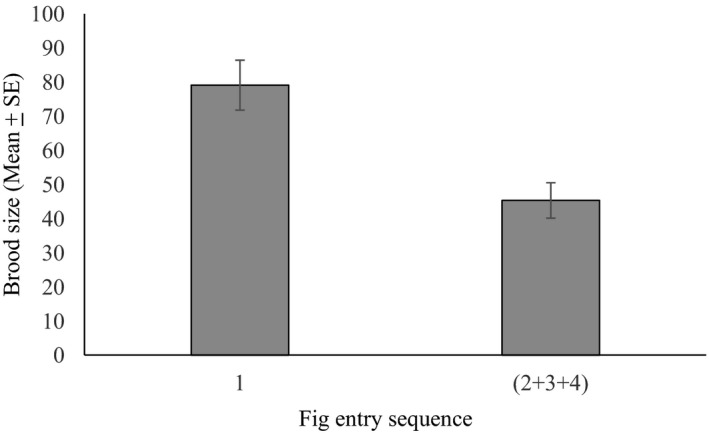
Total brood sizes of *Blastophaga* sp. in the first figs entered by foundresses (including foundresses that did not re‐emerge) and offspring numbers in the figs that were entered subsequently

More offspring were present in the first figs entered by foundresses (mean ± *SE* = 79.11 ± 7.32, *n* = 18 first figs) than in the figs entered by foundresses that had already entered other figs before then (mean ± *SE* = 45.32 ± 5.20, *n* = 22 figs) (GLM, *χ* ^2^ = 185.72, *df* = 1, *p* < .001), based on all foundresses including those that entered just one fig (Figure 3). The mean ± *SE* offspring in the first figs of re‐emerging and non‐re‐emerging foundresses were 78.33 ± 8.84 and 80.70 ± 14.24, respectively, but foundresses that re‐emerged did not have significantly smaller brood sizes in their first figs than foundresses that had all their offspring in one fig (GLM, *χ* ^2^ = 0.27, *df* = 1, *p* > .05). It was not possible to distinguish the sequence of entry among the remaining figs, but the individuals that re‐emerged had larger brood sizes in the first figs they had entered than in the figs they entered later (mean + *SE* for the first figs they entered = 78.33 ± 8.84, *n* = 12 figs compared with 45.32 ± 5.20, *n* = 22 figs) (GLM, *χ* ^2^ = 142.38, *df* = 1, *p* < .001).

The offspring sex ratios of *Blastophaga* sp. were usually strongly female‐biased, with an overall proportion of males of 0.24 (*n* = 2,421 offspring of 18 foundresses, Table 1). Females with all their offspring in a single fig had sex ratios (mean ± *SE*) of 0.16 ± 0.03, which was slightly more female‐biased than the sex ratios of all the offspring of foundresses that re‐emerged (0.26 ± 0.03). This difference in offspring sex ratio was significant (GLM, *χ* ^2^ = 199.32, *df* = 1, *p* < .001), so fewer of the offspring of foundresses that re‐emerged were of direct value to their host plants, because only females can transport pollen.

**FIGURE 4:**
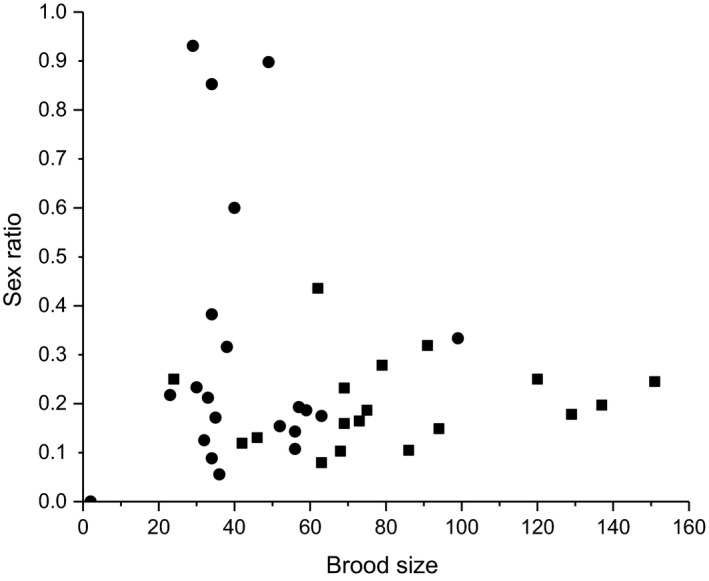
The relationship between offspring sex ratio and brood size in forty figs entered by 18 foundresses. (■) indicates the first figs entered (based on the presence of wings in the ostiole), and (●) indicates the subsequent figs that were entered. Each point indicates the contents of one fig. One foundress generated 1–4 data points

There was a nonsignificant decrease in the extent of female bias among offspring in the subsequently entered figs compared with the first figs they entered (based on all foundresses, including those that only oviposited in one fig, mean ± *SE* = 0.20 ± 0.02 male offspring for the first figs and 0.30 ± 0.06 for subsequent figs) (GLM, *χ* ^2^ = 1.79, *df* = 1, *p* > .05). However, among the twelve foundresses that re‐emerged, four had significantly more female‐biased offspring sex ratio in the first figs they entered than subsequently, and three of these even had more male than female offspring in the subsequent figs (Table 2, Figure 4). There was no significant relationship between offspring sex ratio and brood size in the first fig entered (Spearman's correlation, *r*_s_ = 0.25, *n* = 18, *p* > .05) nor in the subsequent figs entered (Spearman's correlation, *r*_s_ = −0.05, *n* = 22, *p* > .05).

**TABLE 2:** Offspring sex ratios in the first and subsequent figs entered by *Blastophaga* sp. foundress females

Foundress	First fig		Subsequent figs		*χ* ^2^_[1]_	*p*
	Female	Male	Female	Male		
1	35	27	66	33	0.44	.51
2	62	29	2	12	0.001	1.09
3	58	11	48	11	0.16	.69
4	61	14	16	24	20.14	<.001
5	18	6	2	0	0.65	.42
6	106	23	78	10	1.70	.19
7	90	30	23	34	20.1	<.001
8	114	37	70	15	1.49	.22
9	61	12	79	11	0.59	.44
10	58	5	124	21	1.72	.19
11	61	7	86	61	20.93	<.001
12	53	16	46	47	12.47	<.001
Total	777	217	664	279	15.28	<.001

Note

Note that the four individuals displaying significant differences all had a higher proportion of female offspring in the first fig they entered.

## 4 DISCUSSION

The fig wasp pollinators of many species of fig trees enter a single fig to oviposit and so pollinate only the flowers inside that one fig. Such behavior would make very inefficient use of the pollen carried by *Blastophaga* sp. because the female figs of its *F. deltoidea var. angustifolia* host contain far fewer flowers than those of typical *Ficus* species. In practice, the tree is able to make more efficient use of the pollen carried by the female fig wasps because they routinely enter and re‐emerge from several figs. Clearly, the ostioles of its figs remain open for some time after pollinator entry, which provides foundresses with the opportunity to escape from the female figs. The foundresses often opt to do so despite never being able to gain any reproductive advantage from this behavior, as they have lost their wings and have no chance of getting to a male tree. All fig wasps are the progeny of females that entered figs on male, not female trees, and selection on pollinator behavior once inside the figs operates only on male trees. We found that foundresses entering figs on male *F. deltoidea var. angustifolia* also routinely re‐emerged and under our experimental conditions they considerably increased their reproductive success by doing so. Their re‐emergence from figs therefore provides a direct reproductive advantage to the fig wasps, and indirectly benefits both their host male trees (because more of the next generation of pollen‐carrying fig wasp offspring are generated) and the female trees, because the fig wasps replicate this behavior in female figs even though it can be of no benefit to them.

The figs of *F. deltoidea var. angustifolia* are small, and even though its male figs contain far more female flowers than the female figs (94–259 flowers, Mohd Hatta, 2019), there will often have been fewer flowers available for oviposition inside a single fig than the total egg loads of one foundress. A single foundress managed to produce as many as 236 offspring, and so will have required that many female flowers for oviposition, yet an average of less than 150 female flowers were available in any single male fig. Furthermore, under natural conditions many of the figs were entered by more than one foundress, so competition for oviposition sites will have been intense. The abilities of *Blastophaga* sp. foundresses to pollinate and lay eggs in several figs are similar to that of the pollinator of *Ficus montana* Burm. f., which has similarly small figs (Moore et al., 2003; Suleman et al., 2013). Even though carried out under field conditions, unlike the *F. montana* studies, our experimental design will nonetheless have favored those foundresses that opted to re‐emerge because they were sheltered from predators, including ants, as they were walking between figs. The roughly doubling in offspring numbers that they achieved by re‐emergence is therefore likely to be greater than would be achieved under fully natural conditions. Even so, at least some of the wasps that could not lay all their eggs in a single fig will increase their brood size by re‐emerging and entering a second fig.

We may have further overestimated the benefits of re‐emergence for the insects because foundresses that opted to re‐emerge may have had larger egg loads than those that remained in their first figs. These individuals would nonetheless have also been those for which a single fig was likely to offer insufficient oviposition sites.

Two additional factors also have the potential to reduce the extent of the benefits accruing to their hosts by facilitating re‐emergence of pollinator foundresses. The number of seeds generated by the *Kradibia tentacularis* (Grandi) pollinators of *F. montana* drops off rapidly in the second and subsequent figs they enter (Suleman et al., 2013) because of the depletion of their pollen loads (Kjellberg et al., 2014). Pollen depletion is less significant for *F. deltoidea var. angustifolia* because its female figs contain so few flowers that they were almost all pollinated regardless of the number of figs previously entered by an individual foundress. The benefits to male trees of having more fig wasp offspring generated as a result of oviposition in several figs are modified because offspring sex ratios in fig wasps are not fixed, and only pollen‐carrying female offspring are of direct value to the plant (Anstett et al., 1998). Entry into male figs of *F. deltoidea var. angustifolia* by more than one foundress meant that a reduced number of oviposition sites were available to later‐entering foundresses, so their clutch sizes were necessarily smaller. Under these circumstances, male offspring become more valuable to these foundresses because they can mate with the female offspring produced by earlier‐entering foundresses. Changes in offspring sex ratios were observed in single foundress *Blastophaga* sp., even though there was no competition for oviposition sites with other foundresses, with offspring sex ratios tending to be more female‐biased in the first figs they entered, where their clutch sizes were larger. This could be generated by laying mostly male eggs at the start of the oviposition sequences in each fig, as seen in other fig wasps (Suleman et al., 2013), in combination with a resetting of this pattern when females enter subsequent figs. Possible mechanisms for the latter include responding to egg loads (Zhang et al., 2019) or a reduced chance of fertilization associated with the very rapid rate of oviposition that occurs when foundresses first enter a fig (Raja et al., 2008b).

The large seeds of species in the *F. deltoidea* complex may represent an adaptation linked to their epiphytic lifestyles (Berg & Corner, 2005), but hemi‐epiphytic (strangler) figs germinate in similar locations on host trees and establish despite having the small seeds typical of the genus. In the *F. deltoidea* complex, larger seeds have been achieved by reducing the number of flowers per female fig, rather than making the figs larger to accommodate a larger volume of seeds. For the mutualism between fig trees and fig wasps to function, mutual mimicry between the sexes is required at the time that figs are attracting pollinators because of the need to avoid any cues that would allow pollinators to avoid female figs (Grison‐Pigé et al., 2001; Moore et al., 2003). Reflecting this, receptive female figs of *F. deltoidea var. angustifolia* are the same size as the figs on male trees, despite the large difference in the number of flowers they contain. Pollinator re‐emergence has made larger seeds possible despite these fig size constraints, by having fewer larger seeds in each fig while at the same time allowing a single pollinator to pollinate several figs.

Re‐emergence behavior may have been a pre‐adaptation linked to small fig size in an ancestral species (Vereecken et al., 2012). However, within the *F. deltoidea* complex there are varieties with much larger figs than those of *F. deltoidea var. angustifolia*. The male figs of varieties with large figs contain many flowers, and so have many more oviposition sites for the fig wasps, but their female figs still have unusually small numbers of flowers (Mohd Hatta, 2019). The pollinators of these varieties would seem to have less incentive to re‐emerge from the first figs they enter, unless competition for oviposition sites is generated by routinely high numbers of foundresses entering each male fig. Phylogenetic relationships within the complex are unclear (Corner, 1969), and a reliable phylogeny, in combination with comparative behavioral studies, will be needed before the interplay between pollinator behavior and their unusual inflorescence design can be fully understood.

## 5 CONCLUSION

Evolutionary innovations are potentially constrained by the behavior of key mutualists such as obligate pollinators. Almost all figs contain numerous flowers that can utilize the full pollen loads of the fig wasps that enter them to produce hundreds of seeds. Female figs of trees in the *Ficus deltoidea* complex have lost most of these flowers in order to allow for the production of a small number of unusually large seeds inside small figs. A single pollinator can pollinate several of their female figs because they enter figs, re‐emerge, and walk between them. This behavior is of no direct benefit to those individuals, but occurs because of selection that has taken place among earlier generations of fig wasps that were entering figs on male trees.

## CONFLICT OF INTEREST

None declared.

## AUTHOR CONTRIBUTIONS

**Siti Khairiyah Mohd Hatta:** Conceptualization (equal); formal analysis (equal); methodology (lead); writing–original draft (equal); writing–review and editing (equal). **Stephen Compton:** Conceptualization (equal); writing–original draft (equal); writing–review and editing (equal). **Rupert J. Quinnell:** Formal analysis (equal); writing–review and editing (equal). **Abd Ghani Idris:** Methodology (supporting).

### OPEN DATA BADGES

This article has earned an Open Data Badge for making publicly available the digitally‐shareable data necessary to reproduce the reported results. The data is available at https://doi.org/10.5061/dryad.np5hqbzrh.

## Data Availability

Data underlying this work can be assessed through Dryad at https://doi.org/10.5061/dryad.np5hqbzrh
